# Erythema multiforme-like lesions triggered by sun exposure: A case of Rowell syndrome as the initial presentation of systemic lupus erythematosus

**DOI:** 10.1016/j.jdcr.2025.12.032

**Published:** 2025-12-29

**Authors:** Karishma S. Shah, Andrew Creadore, Kenneth Linden, Linda Doan, Michelle S. Min

**Affiliations:** Department of Dermatology, University of California Irvine School of Medicine, Irvine, California

**Keywords:** cutaneous lupus, erythema multiforme, Rowell syndrome, systemic lupus erythematosus

## Introduction

Rowell syndrome (RS) is a rare disease characterized by erythema multiforme (EM)-like lesions in the setting of lupus erythematosus (LE). This association was first described in 1922.^1^ In 1963, Dr. Rowell and his colleagues reported 4 more cases, and cited 2 others, of women with LE and skin lesions resembling EM. Laboratory findings included antinuclear antibodies (ANA) in a speckled pattern and positive rheumatoid factor.^2^

To date, less than 100 cases of EM-like lesions associated with LE have been described in the literature.^3^ Given its rarity, there is question of whether RS is a distinct clinical entity, a coincidence of LE and EM, or on a broader spectrum of LE disease. This can make it challenging to characterize.

## Case report

**Fig 1:**
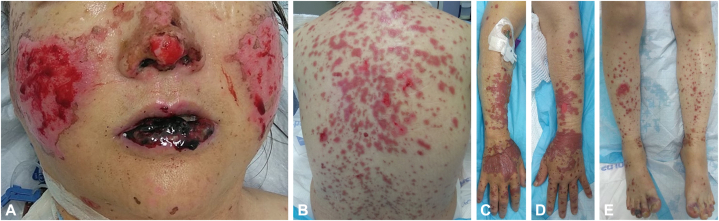
Clinical features of Rowell syndrome upon initial presentation in the emergency department. Flaccid bullae overlying dusky patches in the malar distribution on the face and hemorrhagic erosions of mucosal lips with no evidence of ocular conjunctival erythema or erosions **(A)**. Flaccid bullae overlying dusky erythematous macules and patches on the anterior and posterior trunk and bilateral arms **(B-D)**. Non-palpable petechiae and purpura of bilateral legs and bullae located on dorsal toes **(E)**.

A previously healthy 59-year-old Vietnamese woman presented to the emergency department (ED) with 3 days of dusky blisters and erosions concentrated on her face (forehead, malar cheeks, nose, and mucosal lips with notable sparing of eyelids and nasolabial folds), chest, back, and extremities (worse distally including hands and feet) (Fig 1). Initial laboratory workup revealed thrombocytopenia of 42 thous/mcl and elevated erythrocyte sedimentation rate and C-reactive protein.

**Fig 2:**
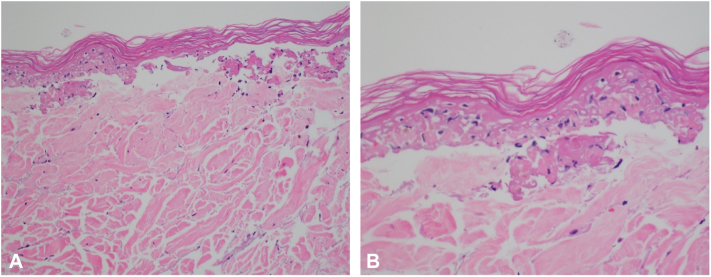
Permanent punch biopsy from left calf. Interface dermatitis with necrotic keratinocytes, subepidermal split and focal areas of near full thickness necrosis at 20× **(A)** and 40× magnification **(B)**.

A fresh frozen shave biopsy from her left shoulder and punch biopsy from her left calf revealed a subepidermal split, vacuolar interface dermatitis, and focal areas of near full-thickness epidermal necrosis. No prominent peri-adnexal inflammation or increased dermal mucin were noted (Fig 2).

Given histopathologic findings, toxic epidermal necrolysis or EM was favored. However, no culprit medication or infectious source could be identified. Herpes simplex and varicella zoster virus polymerase chain reaction tests from the lower lip and mycoplasma IgM/IgG serologies all returned negative. Based on the working diagnosis, cyclosporine 5 mg/kg daily was initiated pending further workup, and the patient’s lesions began to heal.

Eventually, ANA testing returned positive at >1:320 titer with speckled pattern, and the patient was referred to a rheumatologic-dermatology specialist. Further review of systems was notable for diffuse alopecia and arthralgias. Importantly, the patient recalled being at the beach without sun protection a few days prior to presenting to the ED. A diagnosis of RS was suspected, later supported by positive Sjogren's-syndrome-related antigen A (SSA) and Smith antibodies. Collagen VII antibodies via enzyme-linked immunosorbant assay were negative. Cyclosporine was discontinued in the setting of acute kidney injury and diagnostic insight. The patient was then transitioned to hydroxychloroquine (HCQ) and methotrexate (MTX), with continued healing of EM-like lesions.

**Fig 3:**
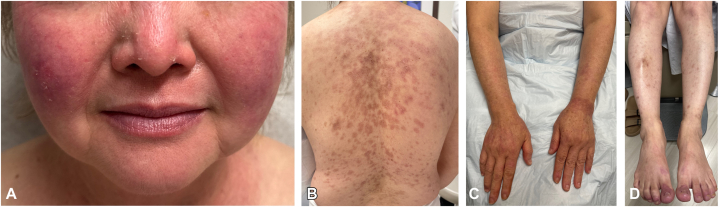
Clinical features at outpatient follow-up 4 months after initial presentation to the emergency department. Numerous re-epithelialized hyperpigmented *pink macules* and patches on the face **(A),** central trunk **(B),** and upper and lower extremities **(C, D)** with sun exposed areas more involved than sun protected areas.

Within 2 weeks, our patient endorsed significant improvement. The only remaining active areas were refractory chilblains on her toes for which tadalafil was also initiated with good effect. At most recent follow-up 4 months later, her skin has remained clear (Fig 3). There are no longer signs of active systemic lupus erythematosus (SLE). She continues to remain on HCQ, MTX, and tadalafil.

## Discussion

RS was first described as an association between EM and LE. Based upon review of the literature, the median age at diagnosis is 32 years, with the third and fourth decades being the most common. Interestingly, in a large majority, LE diagnosis precedes, sometimes by many years, the appearance of EM-like lesions.^3^ Thus, our patient highlights a unique case in which an eruption of EM-like lesions was the initial presenting symptom of LE at twice the typical age.

In 1963, Rowell et al. proposed original diagnostic criteria: LE, EM-like lesions (with absence of any known precipitating factors), speckled pattern of ANA, antibodies against saline extracts of human tissue, and positive rheumatoid factor.^2^ Since then, multiple iterations of diagnostic criteria have been proposed. In 1995, Lee et al suggested including chilblains and anti-La/SSB antibody.^4^ Zeitouni et al reported 2 new cases of RS and proposed major and minor criteria, with 3 major and 1 minor criteria necessary for diagnosis.^5^ The major criteria are as follows: SLE, discoid lupus erythematosus, or SCLE; EM-like lesions; speckled pattern of ANA. The minor criteria are chilblains; anti-Ro/SSA or anti-La/SSB; and positive rheumatoid factor.

More recently in 2020, Torchia et al systematically reviewed cases and found a difference between EM-like lesions in subacute cutaneous LE (SCLE)/acute cutaneous LE (ACLE) and chronic cutaneous LE (CCLE). They concluded that in the setting of SCLE/ACLE, most EM-like lesions are morphologic variants of LE-specific skin lesions. However, they reported that EM-like lesions in the setting of CCLE had a distinctive combination of features and suggested maintaining the term “Rowell syndrome.” Proposed RS major criteria were as follows: presence of CCLE (DLE and/or chilblain); EM-like lesions; at least 1 positivity among speckled ANA, anti-Ro/SSA, anti-La/SSB antibodies; and negative direct immunofluorescence on lesional EM-like lesions. Minor criteria were absence of infectious or pharmacologic triggers; absence of typical EM location (acral and mucosal); and presence of at least 1 additional criterion for diagnosis of SLE excluding photosensitivity, malar rash, and oral ulcers. Presence of all 4 major criteria and at least 1 minor criterion is required.^3^

Unfortunately, there is less consistency in RS’s histologic findings. Some patients have biopsy results consistent with EM, showing full thickness necrosis of the overlying epidermis, necrotic keratinocytes, and sparse perivascular dermal lymphocytic infiltrate.^5^, ^6^, ^7^ Patients might or might not have histopathologic features of cutaneous lupus, such as perivascular lymphocytic infiltrates and mucin deposition between collagen bundles in early stages or epidermal atrophy and dermal fibrosclerosis in late stages.^8^ Therefore, we concur that histologic findings are not critical to diagnostic criteria, as described above.

Regardless of which diagnostic criteria are applied, our patient met RS diagnosis based on EM-like lesions, positive ANA with speckled pattern, anti-SSA antibody, chilblains, and absence of infectious or pharmacologic triggers. Anti-Smith, thrombocytopenia, alopecia, arthralgias, and photosensitivity/malar rash also helped our patient meet the diagnosis of SLE for the first time.

Overall, our case demonstrates the need to be suspicious of RS in a case that mimics EM when lesions favor photo-exposed regions, even in a patient with no previous diagnosis of LE and past the typical age of presentation. If RS is being considered, clinicians should inquire of recent sun exposure. Furthermore, if considering RS in the setting of SLE, though effective, cyclosporine should be administered cautiously given its established nephrotoxicity.^9^^,^^10^

## Conflicts of interest

None disclosed.
